# Rapid single-colony whole-genome sequencing of bacterial pathogens

**DOI:** 10.1093/jac/dkt494

**Published:** 2013-12-25

**Authors:** Claudio U. Köser, Louise J. Fraser, Avgousta Ioannou, Jennifer Becq, Matthew J. Ellington, Matthew T. G. Holden, Sandra Reuter, M. Estée Török, Stephen D. Bentley, Julian Parkhill, Niall A. Gormley, Geoffrey P. Smith, Sharon J. Peacock

**Affiliations:** 1 Department of Medicine, University of Cambridge, Cambridge, UK; 2 Clinical Microbiology and Public Health Laboratory, Public Health England, Cambridge, UK; 3 Illumina United Kingdom, Great Chesterford, UK; 4 Wellcome Trust Sanger Institute, Hinxton, UK; 5 Cambridge University Hospitals NHS Foundation Trust, Cambridge, UK

**Keywords:** infectious diseases, antibiotic resistance, typing

## Abstract

**Objectives:**

As a result of the introduction of rapid benchtop sequencers, the time required to subculture a bacterial pathogen to extract sufficient DNA for library preparation can now exceed the time to sequence said DNA. We have eliminated this rate-limiting step by developing a protocol to generate DNA libraries for whole-genome sequencing directly from single bacterial colonies grown on primary culture plates.

**Methods:**

We developed our protocol using single colonies of 17 bacterial pathogens responsible for severe human infection that were grown using standard diagnostic media and incubation conditions. We then applied this method to four clinical scenarios that currently require time-consuming reference laboratory tests: full identification and genotyping of salmonellae; identification of *bla*_NDM-1_, a highly transmissible carbapenemase resistance gene, in *Klebsiella pneumoniae*; detection of genes encoding staphylococcal toxins associated with specific disease syndromes; and monitoring of vaccine targets to detect vaccine escape in *Neisseria meningitidis*.

**Results:**

We validated our single-colony whole-genome sequencing protocol for all 40 combinations of pathogen and selective, non-selective or indicator media tested in this study. Moreover, we demonstrated the clinical value of this method compared with current reference laboratory tests.

**Conclusions:**

This advance will facilitate the implementation of whole-genome sequencing into diagnostic and public health microbiology.

## Introduction

Standard methods used in diagnostic and reference microbiology laboratories to characterize bacterial pathogens can be time-consuming and complex.^1,2^ Once a bacterial pathogen is isolated on a primary culture plate, it has to be identified to provide an early prediction of the potential disease progression and to identify antibiotics to which the pathogen is intrinsically resistant. This is followed by antibiotic susceptibility testing to identify acquired drug resistances and, in some cases, the elucidation of significant resistance mechanisms. The detection of virulence determinants such as toxins or antigens can also inform clinical management.^1,2^ Finally, bacterial typing may be required for a limited number of isolates to support infection control and outbreak investigations.^1,2^

In contrast to this multifaceted testing algorithm, rapid benchtop sequencers can provide multiple pieces of clinically relevant information in a single process.^3–11^ The starting material for bacterial whole-genome sequencing (WGS) is typically purified DNA extracted from liquid culture. In a clinical setting, however, it is important to sequence directly from a single bacterial colony on primary isolation plates.^2,9,12^ First, this avoids the delays and costs that result from subculture and DNA extraction.^4,5,13^ This is vital to direct early infection control interventions to contain outbreaks and, in the case of foodborne outbreaks, to maximize the possibility of recovering organisms from the presumed source.^7,8,14^ Second, it minimizes the chance of contamination (with either a different species or a different variant of the same species) associated with subculture or picking multiple colonies from a primary isolation plate.^7^ Third, it reduces the chance of introducing genomic changes, such as the loss of unstable plasmids.^15,16^ Our aim was therefore to develop a simple protocol to enable WGS directly from a single bacterial colony and to validate it using a broad range of pathogenic bacteria.

## Materials and methods

### Ethical approval

Ethical approval was not required as clinical isolates were collected, processed and stored as part of routine clinical care at the Cambridge Public Health England Clinical Microbiology and Public Health Laboratory. Clinical isolates were anonymized prior to DNA extraction and sequencing. Research and development (R&D) approval for WGS of anonymized bacterial isolates was granted by the Cambridge University Hospitals NHS Foundation Trust R&D Department.

### Reference species and clinical isolates

Details of the 17 reference and 2 clinical isolates, which encompass a diverse range of bacterial species with different modes of transmission that occur both in the hospital and community setting, are shown in Table 1 and Tables S1 and S2 (both available as Supplementary data at *JAC* Online).

**Table 1.** Overview of the 17 reference strains used in this study, which cause significant disease in the UK and globally;^a^ in addition, we studied two clinical isolates (one *Salmonella* Enteritidis and one *K. pneumoniae*)

Species	Gram classification	Chromosome size (Mb)	GC content (%)	No. of plasmids^b^
*Acinetobacter baumannii* ATCC 17978	−	3.98	39	2
*Campylobacter jejuni* subsp. *jejuni* NCTC 11168	−	1.64	31	
*Clostridium difficile* 630	+	4.29	29	1
*Enterobacter cloacae* subsp. *cloacae* type strain ATCC 13047	−	5.31	55	2
*Enterococcus faecalis* V583	+	3.22	38	3
*Escherichia coli* str. K-12 substr. MG1655	−	4.64	51	
*Haemophilus influenzae* Rd KW20	−	1.83	38	
*Klebsiella pneumoniae* subsp. *pneumoniae* MGH 78578	−	5.32	57	5
*Legionella pneumophila* subsp. *pneumophila* str. Philadelphia 1	−	3.40	38	
*Neisseria meningitidis* serogroup B strain MC58	−	2.27	52	
*Pseudomonas aeruginosa* PAO1	−	6.26	67	
*Salmonella enterica* subsp. *enterica* serovar Enteritidis str. P125109	−	4.69	52	1
*Shigella sonnei* 53G	−	4.99	51	4
*Staphylococcus aureus* HO 5096 0412	+	2.83	33	1
*Streptococcus agalactiae* 2603V/R	+	2.16	36	
*Streptococcus pneumoniae* TIGR4	+	2.16	40	
*Streptococcus pyogenes* M1	+	1.85	39	

^a^Tables S1 and S2, available as Supplementary data at *JAC* Online.

^b^The sizes and GC contents of the plasmids can be found in Table S2 (available as Supplementary data at *JAC* Online).

### Culture conditions

All bacterial isolates were grown from frozen stocks (stored at −80°C) as specified in Table S3 (available as Supplementary data at *JAC* Online).

### DNA extraction, library preparation and WGS

Mechanical lysis with 425–600 micron glass beads (Sigma–Aldrich, St Louis, MO, USA) was used to extract DNA (Figure S1, available as Supplementary data at *JAC* Online). Libraries were prepared using one of two kits, depending on the colony size. The standard Nextera kit (Illumina, San Diego, CA, USA),^17^ which requires 50 ng of DNA in 25 μL of UltraPure DNase RNase-free distilled (UP) water (Invitrogen, Paisley, UK), was used for species that formed large colonies (defined as a diameter of ≥1.5 mm), and the Nextera XT kit (Illumina), which needs only 1 ng of DNA in 5 μL, was used for colonies with a smaller diameter (Table S4, available as Supplementary data at *JAC* Online). To obtain sufficiently concentrated DNA after lysis, the amount of UP water used for the lysis step was varied depending on the pathogen. For the Nextera kit, a single colony, or part of a single colony if the colony in question was very large (i.e. several millimetres) was picked with a 1 μL loop and resuspended in 40 μL of UP water in a 1.5 mL screw-capped Eppendorf tube containing glass beads [at a ratio of 1 : 3 of beads relative to water to minimize the amount of ‘dead’ volume (beads were transferred using the cap of a 0.2 mL PCR tube)]. The sample was vortexed at speed 6 on a Vortex-Genie with a microtube foam insert (Scientific Instruments, Bohemia, NY, USA) for 5 min and subsequently centrifuged in a benchtop centrifuge for 2 min at full speed. Twenty-five microlitres of the supernatant was removed and its DNA concentration was quantified using the Qubit dsDNA HS Assay Kit (Invitrogen, Eugene, OR, USA) and diluted with UP water to obtain 50 ng of DNA in 25 μL.

For small colonies processed with the Nextera XT kit (Table S4, available as Supplementary data at *JAC* Online), the process was similar. A single colony was resuspended in 25 μL of UP water with beads, then vortexed and centrifuged as described earlier, except that only 10 μL of supernatant was removed. DNA was quantified and diluted to obtain 1 ng in 5 μL of UP water. For *Clostridium difficile* 630 and *Streptococcus pneumoniae* TIGR4, which yielded the lowest amounts of DNA, the initial resuspension volume was reduced to 15 μL to obtain sufficiently concentrated DNA (7 μL of the lysed supernatant were removed in this case after lysis). Care was taken to dilute the DNA accurately, which meant that if the supernatant was too concentrated (i.e. if <0.5 μL of supernatant would have had to be transferred) an additional dilution step was done to ensure that 1 ng of DNA could be pipetted accurately.

Both the diluted and undiluted supernatants were kept on ice and the DNA was immediately processed with the respective Nextera kit. The tagmentation was also set up on ice. The manufacturer's protocol was used with the following exceptions for the standard Nextera kit. Given that the solution with the tagmented DNA probably contained viable bacteria, it was cleaned up with individual capped DNA Clean & Concentrator columns (Zymo Research, Irvine, CA, USA) rather than the 96-well version of the kit. Eppendorf tubes (1.5 mL) were used to mix the binding buffer and for the elution step. Moreover, the Zymo column was placed onto a new 2 mL collection tube (Qiagen, Hilden, Germany) after each spin and the old tube discarded. For both Nextera kits, capped 0.2 mL PCR strip tubes were used for the tagmentation and amplification steps instead of 96-well plates. Rather than cleaning up 50 μL of PCR product as specified in the protocol, 40 μL of product were added to 10 μL of UP water for this step to compensate for evaporation and pipetting errors during the PCR amplification step.

The final libraries were quantified as described earlier and 1 μL of the undiluted library was analysed on a 2100 Bioanalyzer using a High Sensitivity DNA chip (Agilent Technologies, Waldbronn, Germany; Table S4, available as Supplementary data at *JAC* Online). Sequencing was done on the MiSeq sequencing platform (Illumina) using 150 bp paired-end reads as previously described.^5^ The sequence data were deposited at the European Nucleotide Archive (Table S2, available as Supplementary data at *JAC* Online).

The clinical New Delhi metallo-β-lactamase (NDM)-1-producing *Klebsiella pneumoniae* was highly mucoid and care was taken to transfer biomass from the centre of the colony into 40 μL of UP water. After vortexing and centrifugation, 10 μL of the supernatant were removed slowly to minimize carryover of extracellular components. The supernatant was cleaned up using the DNA Clean & Concentrator kit as described earlier and the DNA was eluted in 10 μL of UP water, which was then processed with the Nextera XT kit.

Where appropriate, the aforementioned steps were done in a Microflow Class I Advanced Biosafety Cabinet (Bioquell UK, West Portway, UK).

### Sterility tests

To check for bacterial cell death during the single-colony library preparation, three libraries for each of the 40 combinations of pathogen and growth conditions (Tables S3 and S4, available as Supplementary data at *JAC* Online) were either incubated in 10 mL of brain heart infusion broth (Public Health England Media Services, London, UK) for 5 days in air at 36°C (except for *S. pneumoniae* and *Neisseria meningitidis*, which were incubated with 5% CO_2_), or on solid medium for the following: *Clostridium difficile* was incubated anaerobically on Brazier's CCEY agar and fastidious anaerobe agar with horse blood (Oxoid, Basingstoke, UK) for 5 days at 36°C; *Haemophilus influenzae* Rd KW20 was incubated on Columbia agar with chocolate horse blood for 5 days at 36°C with 5% CO_2_; *Campylobacter jejuni* was incubated on Columbia blood agar with horse blood in a microaerophilic environment at 42°C for 10 days; and *Legionella pneumophila* was incubated on *Legionella* BMPA selective agar for 10 days at 36°C with 5% CO_2_. More details about the culture media can be found in Table S3 (available as Supplementary data at *JAC* Online).

### Analysis of reference genomes

The sequence data of the 17 reference isolates were analysed using the default settings of the ‘resequencing’ workflow in the Illumina Sequence Integration Software (ISIS) version 2.0, which is installed by default on the MiSeq instrument. The software and documentation of the latest version of the software are available on the Illumina customer support web site (http://support.illumina.com/sequencing/sequencing_instruments/miseq/downloads.ilmn).

The following steps are automatically performed by the software: demultiplexing; FASTQ generation and adapter trimming; alignment of the reads against the reference genome [by concatenating chromosomes and plasmids when relevant (Table S2, available as Supplementary data at *JAC* Online)]; and variant calling. ISIS uses the Burrows–Wheeler Aligner^18^ for paired-reads alignment with its default parameters, apart from read trimming of bases with <Q15. ISIS uses the GATK UnifiedGenotyper^19,20^ for calling both single-nucleotide polymorphisms (SNPs) and insertions/deletions (indels) using its default parameters, except for also outputting variants with phred-scaled confidence between 10 and 30 flags as LowQual.

SAMTools^21^ was used to calculate the following metrics for Table S5 (available as Supplementary data at *JAC* Online): the proportion of unaligned reads per sample; the average depth of coverage per reference sequence; the proportion of reference positions that were not covered by at least one read; the proportion of reference positions that had >10× coverage; and the SNPs and indels that passed filtering with GATK caller and had a variant base frequency of 100%.

### Retrospective analysis of clinical isolates

To confirm the genetic basis of carbapenem resistance in the clinical *K. pneumoniae* isolate, we used the same set of carbapenemases tested by the Public Health England Antimicrobial Resistance and Healthcare Associated Infections Reference Unit (AMRHAI, London, UK). Moreover, we included *bla*_GES-1_ (Table S6, available as Supplementary data at *JAC* Online). The genome of the isolate in question was assembled *de novo* using Velvet^22^ and reference resistance genes were then mapped onto the assembly using SMALT software (http://www.sanger.ac.uk/resources/software/smalt/), allowing the same gene to map multiple times to the assembly using a cut-off for detection of 90% DNA sequence identity. To further assess whether the candidate genes identified from the assembly were present, the raw sequencing reads were mapped to each candidate gene, allowing the assessment of coverage and SNP/indel variation in the isolate of interest. The *bla*_NDM-1_ hit was confirmed using a BLAST search, and no amino acid changes compared with *bla*_NDM-1_ were found. The same approach was used for the detection of toxin genes in methicillin-resistant *Staphylococcus aureus* (MRSA), as previously described.^5^

For the analysis of the clinical *Salmonella* isolate, we constructed a maximum-likelihood tree of core genome SNPs using RAxML^23^ and Fast tree.^24^ The core genome was identified using comparative genomic analysis, and excluded mobile genetic elements and genomic island regions from the *Salmonella* Enteritidis P4 chromosome.^25^ The paired-end reads from the study isolate, and DNA sequence from other previously sequenced *Salmonella* isolates (Table S7, available as Supplementary data at *JAC* Online), were mapped against the chromosome of *Salmonella* Enteritidis PT4.^25^ SNPs were identified as previously described.^26^

## Results and discussion

### Assay development and validation

We initially explored whether the observation by Adey *et al*.,^17^ who found that it is possible to generate a sequencing library directly from *Escherichia coli* colonies, could be reproduced with other Gram-negative bacteria as well as Gram-positive bacteria such as MRSA. We found that simply picking a colony of MRSA into a PCR tube containing sterile water and vortexing it before adding Nextera reagents generated successful genomic libraries. By contrast, the use of lysozyme, which is often used to lyse Gram-positive bacteria, inhibited the formation of DNA libraries (data not shown). In our final protocol we therefore opted for mechanical lysis using glass beads, which can be used for any bacterial species, followed by a dilution step of the supernatant to control the insert size of the final libraries [by normalizing the amount of DNA relative to the number of transposons (Figure S1, available as Supplementary data at *JAC* Online)].

We evaluated our protocol using 17 bacterial pathogens that are designated as ‘alert organisms’ by the UK Department of Health or are subject to surveillance in England [Table 1 and Table S1 (available as Supplementary data at *JAC* Online)] and internationally. We selected one representative isolate for each species using the criteria that it had been sequenced previously and represented a clinically relevant strain. For example, for *N. meningitidis* we used the serogroup B strain MC58 because this is the most prevalent serogroup in the UK and was used to develop the 4CmenB vaccine.^27^ The genomes of the 17 bacterial species had a range of sizes and GC contents, and up to five plasmids [Table 1 and Table S2 (available as Supplementary data at *JAC* Online)].

To ensure that our DNA extraction protocol was geared for use in routine diagnostic laboratory practice, we grew the reference isolates using the media and incubation conditions specified in the standard operating procedures for the isolation of each species from clinical specimens at the Cambridge Public Health England Clinical Microbiology and Public Health Laboratory (Table S3, available as Supplementary data at *JAC* Online).^1^

Irrespective of the species, colony size or culture medium, we found that suspending all or part of a single colony in water followed by vortexing with glass beads for 5 min yielded sufficient DNA to successfully prepare standard Nextera or Nextera XT libraries, as judged by their concentration and size distribution (Table S4, available as Supplementary data at *JAC* Online). Importantly, this method did not require any additional clean-up steps prior to library preparation, with the exception of a highly mucoid clinical isolate of *K. pneumoniae* (whether this also applies to other mucoid isolates remains to be determined). Moreover, the libraries were found to be sterile, allowing subsequent handling in a Containment Level 1 laboratory (please refer to the Supplementary data for a more detailed discussion of this aspect).

We further confirmed the quality of the single-colony protocol by sequencing one library for each of the 17 reference isolates using the Illumina MiSeq platform. On average, 94% of each chromosome and plasmid had coverage of >10×, which represents the minimum commonly used to call SNPs (Table S5, available as Supplementary data at *JAC* Online).^15^ Those plasmids that showed <90% coverage had poor mapping due to the presence of homologous regions elsewhere in the genome.^28^ A single plasmid from *Shigella sonnei* 53G was not covered, but further investigation determined that this plasmid was not present in our stock of this isolate. The differences (SNPs and indels) that we found relative to the published references probably represent errors in the reference genomes or minor differences between variants of the reference strains used in different laboratories (Table S5, available as Supplementary data at *JAC* Online).^29,30^

### Application to four clinical scenarios

To illustrate the potential value of rapid single-colony WGS for diagnostic purposes, we applied our protocol to four clinical scenarios in which bacterial isolates are often sent to a reference laboratory: (i) full identification of a clinical *Salmonella* isolate; (ii) verification of carbapenem resistance in a *K. pneumoniae* isolate; (iii) detection of toxin genes in MRSA; and (iv) the investigation of antigens in the vaccine strain of *N. meningitidis*. Each can result in long delays compared with diagnostic functions that are performed locally.

**Figure 1.**
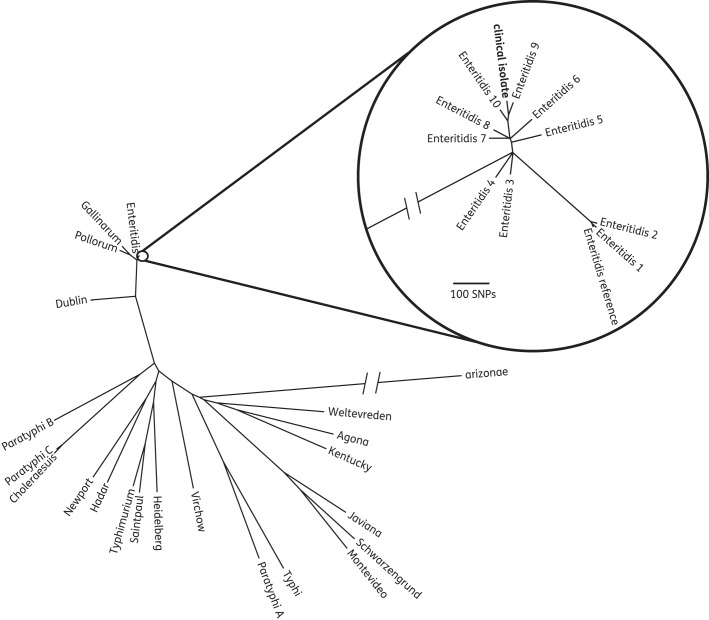
Unrooted maximum-likelihood tree of the clinical *Salmonella* isolate in the context of 21 different *S. enterica* serovars (e.g. Dublin) and *S. enterica* subsp. *arizonae* (with *Salmonella* Enteritidis PT4 as reference^25^). This not only showed that the clinical isolate belonged to the serovar Enteritidis, which matched the reference laboratory results, but simultaneously provided genome-level discrimination between isolates (i.e. the study isolate was only distantly related to the other previously sequenced Enteritidis isolates). Thus single-colony WGS of salmonellae could not only replace serotyping but all currently used epidemiological techniques.

Bacterial pathogens are usually identified at a species or genus level, which can be achieved in laboratories using biochemical tests or matrix-assisted laser desorption/ionization time-of-flight mass spectroscopy (MALDI-TOF).^2^ For some organisms, however, this level of discrimination is insufficient for clinical management. Salmonellae, for example, are most often differentiated into serovars at reference laboratories using several agglutination reactions.^31^ This extra step discriminates between those species associated with typhoid fever versus non-typhoidal gastroenteritis, and sometimes gives clues as to the possible source (e.g. if a rare serovar associated with pets such as reptiles is identified^32^). We randomly selected a clinical isolate of *Salmonella* cultured from faeces in our diagnostic laboratory, which had been identified by the reference laboratory using agglutination methods as *Salmonella enterica* subsp. *enterica* serovar Enteritidis. WGS was performed using the single-colony protocol, and the sequence placed into a phylogenetic tree containing several previously sequenced *S. enterica* serovars (Figure 1). We confirmed from the position in the tree that the test isolate was serovar Enteritidis, and simultaneously provided genome-level discrimination between isolates.

**Figure 2.**
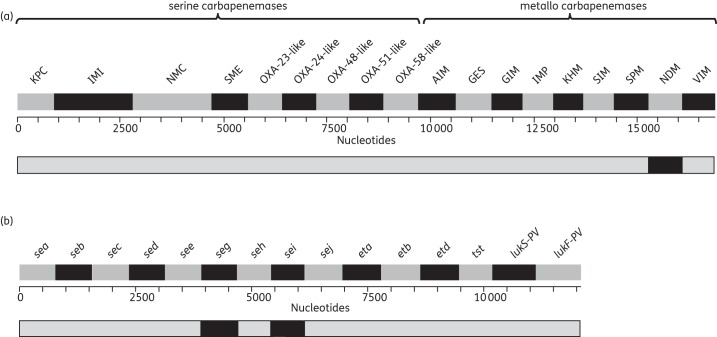
Heat maps showing the presence (black) or absence (grey) of resistance or virulence determinants. (a) Carbapenemase genes for the clinical *K. pneumoniae* isolate. (b) Toxome for MRSA reference strain.

For antimicrobial susceptibility testing, a single inexpensive front-line assay (e.g. a disc diffusion test or automated liquid-culture susceptibility assay) is usually able to detect the most common phenotypic resistance patterns within 24 h.^2,5^ However, to confirm or refute certain significant resistance mechanisms (e.g. the presence of the gene encoding NDM, a carbapenemase that is highly transmissible and therefore demands enhanced infection control procedures) requires the use of one or more secondary assays in a local laboratory, which have to be complemented with further time-consuming reference laboratory tests.^9,33^ By contrast, single-colony WGS enabled the gold-standard molecular detection of the *bla*_NDM-1_ gene in a clinical isolate of *K. pneumoniae* that was isolated from a perinephric abscess (Figure 2a).^32,34^ This agreed with the PCR results obtained by the AMRHAI, where this strain had been analysed after first isolation.

Detection of bacterial genes encoding virulence determinants is not undertaken commonly, but the AMRHAI offers detection of a panel of 14 *S. aureus* toxin genes using multiplex PCRs in cases where toxic shock syndrome is suspected in a patient. We found that the equivalent information could be derived from our MRSA reference strain using the single-colony protocol (Figure 2b). We also had full coverage of all six genes encoding antigens used in the 4CmenB vaccine (fHbp, GNA2091, NHBA, GNA1030, NadA and PorA^27^) in the WGS data of the *N. meningitidis* reference strain (data not shown), which could be used to monitor the effects of the introduction of 4CmenB on these antigens.^35^

### Concluding remarks

Next-generation sequencing is possible directly from clinical samples.^36,37^ Yet in light of the low rate of positivity of most clinical samples, and the fact that culture and PCR are usually more sensitive than WGS, this approach is not cost-effective in most clinical scenarios.^2^ As a result, a primary culture step will remain routine in diagnostic bacteriology in the foreseeable future.^2,38^ Our sample preparation method does, however, eliminate the need for a secondary culture and manual DNA extraction with a kit for 17 of the most clinically important bacterial pathogens, provided that a single colony is recovered on the primary isolation plate (i.e. primary plates that show evidence of mixed, overlapping growth would still need to be subcultured in order to recover pure, single colonies for phenotypic susceptibility testing and WGS). This represents a major advance given that subculture can exceed the time required for library preparation and WGS using rapid benchtop sequencers.^2,9,12^

Once the analysis of sequence data has been fully automated to generate reports suitable for clinicians, and the cost of sequencing has fallen further, we predict that rapid single-colony WGS will solve one of the major problems facing diagnostic bacteriology.^1,2,9,39,40^ Because the four clinical scenarios addressed in this paper are relatively rare in the UK, the costs for reagents, equipment and staff for the associated tests are often prohibitive for local diagnostic laboratories.^9^ By contrast, combining different bacterial species in the same sequence run will probably make single-colony WGS cost-effective in the future. As a result, local laboratories will be able to provide many current reference laboratory tests, which would avoid the delays associated with shipping.^2,9^ In fact, WGS can address multiple clinically relevant questions for many pathogens, as illustrated in this proof-of-principle study. For example, we showed that WGS of salmonellae can replace serotyping as well as current typing techniques such as multilocus sequence typing, variable-number tandem repeat analysis, PFGE and microarrays.^14,31,39^ Similarly, the WGS data from the NDM-1-producing *K. pneumoniae* that we used to confirm the carbapenem resistance mechanism could equally have been used to track onward transmission^9,16,41^ or to monitor the genetic basis of resistance to other antibiotics, thereby providing a powerful surveillance tool.^2,5,42–44^

## Funding

This work was supported by a grant from the UK Clinical Research Collaboration Translational Infection Research Initiative, Medical Research Council (G1000803). Contributions were also received from: the Biotechnology and Biological Sciences Research Council, National Institute for Health Research (NIHR), on behalf of the UK Department of Health and the Chief Scientist Office of the Scottish Government Health Directorate; Public Health England; the Cambridge Biomedical Research Centre, NIHR (to S. J. P.); and the Wellcome Trust Sanger Institute (to J. P.). C. U. K. is a Junior Research Fellow at Wolfson College, Cambridge.

## Transparency declarations

L. J. F., A. I., J. B., N. A. G. and G. P. S. are employees and shareholders of Illumina Inc. M. J. E. and M. E. T. were funded to attend conferences by Bruker Daltonics and Illumina Inc., respectively. M. E. T. has received speaker's honoraria and book royalties from Oxford University Press. J. P. has received funding for travel and accommodation from Pacific Biosciences Inc. and Illumina Inc. S. J. P. is a consultant for Pfizer Inc. and received funding for travel and accommodation from Illumina Inc. All other authors: none to declare.

## Supplementary data

Supplementary data, including Figure S1 and Tables S1 to S7, are available at *JAC* Online (http://jac.oxfordjournals.org/).

Supplementary Data
