# Supplementary material for: Rapid single-colony whole-genome sequencing of bacterial pathogens
Source: J Antimicrob Chemother. 2013 Dec 25;69(5):1275–81. doi: 10.1093/jac/dkt494 (PMC3977605; doi:10.1093/jac/dkt494)
Supplement: Supplementary Data [file supp_69_5_1275__index.html]

Rapid single-colony whole-genome sequencing of bacterial pathogens — Supplementary Data 

# Rapid single-colony whole-genome sequencing of bacterial pathogens

## Supplementary Data

Supplementary Data

**Files in this Data Supplement:**

- Supplementary data - doc file
