# Supplementary material for: Rapid single-colony whole-genome sequencing of bacterial pathogens
Source: J Antimicrob Chemother. 2013 Dec 25;69(5):1275–81. doi: 10.1093/jac/dkt494 (PMC3977605; doi:10.1093/jac/dkt494)
Supplement: Supplementary Data [file supp_dkt494_dkt494supp_data.doc]

**Supplementary data**

**Biosafety considerations**

We used this protocol for known hazard group (HG) 1 or 2 organisms, which encompass the vast majority of bacteria isolated in the UK, and therefore focused on demonstrating that DNA libraries were sterile after the PCR clean-up step to allow further processing in a containment level (CL) 1 environment (i.e. we did not investigate at which point exactly the samples became sterile). Potential users of this method should complete appropriate risk assessments and biosafety experiments before using or adapting the single-colony protocol. This is particularly important for HG3 or suspected HG3 organisms, which have to be cultured in a CL3 laboratory and fully inactivated prior to their removal to CL1 or 2 environments. In this context, it may be impractical to perform any of the enzymatic library preparation steps in a CL3 laboratory. Instead, users could add a heating step after the mechanical lysis with beads to inactivate the organism in question before performing the remaining steps of the single-colony protocol outside of a CL3 laboratory. However, care has be taken to avoid extensive DNA degradation, which is incompatible with the Nextera library preparation method.[^1^](#_ENREF_1)

**Figure S1**

Schematic outlining the protocol for single-colony WGS using the Nextera XT kit (for reference species that formed large colonies (i.e. those with a diameter of at least 1.5 mm) we used the standard Nextera kit, but we expect that the XT kit could have been used as an alternative). For the mucoid NDM-1-producing *K. pneumoniae* isolate a cleanup step had to be added after bead lysis.


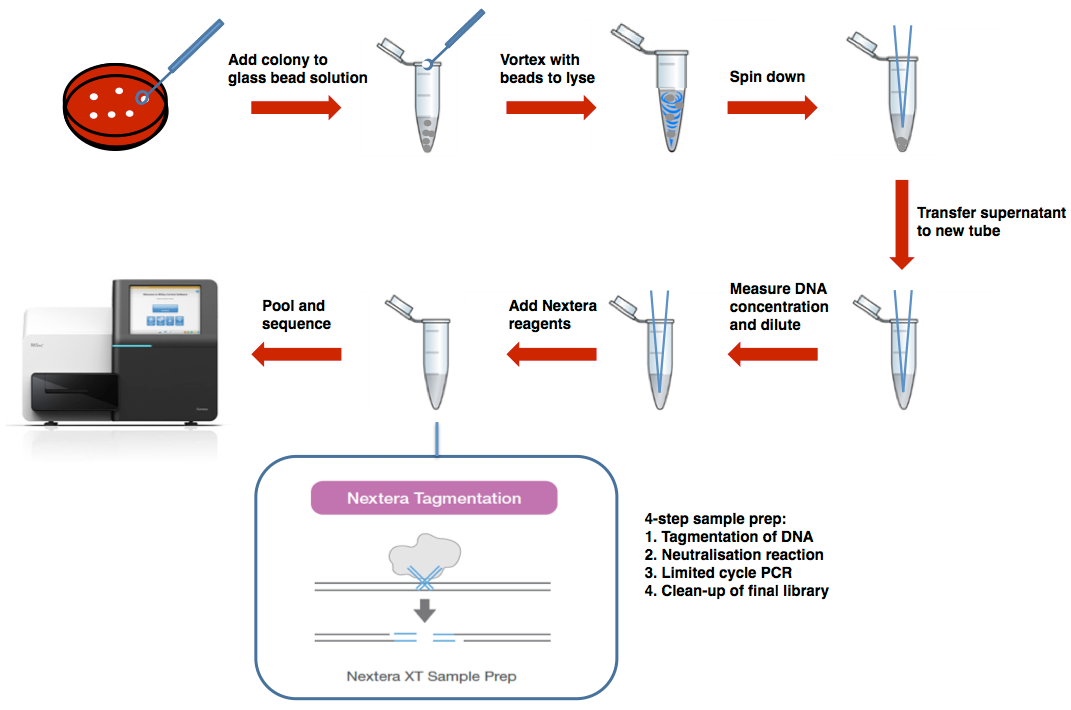


**Table S1**

**Overview of the prevalence of the 17 bacterial species or diseases included in this study and the potential role of WGS in their diagnosis. Most of these pathogens are either designated as ‘alert organisms’ by the UK Department of Health or are subject to mandatory surveillance programs.**[**^2-4^**](#_ENREF_2) **We only included pathogens for which single colonies are routinely isolated in our diagnostic laboratory (e.g. *Mycobacterium tuberculosis* was excluded as it is normally isolated in liquid culture in the UK). Depending on the pathogen in question, the cost of rapid WGS could be justified for specialist identification (ID) of a pathogen or toxin detection, to identify vaccine escape mutants by monitoring the appropriate antigens, or to investigate significant resistance mechanisms such as carbapenem resistance, which cannot be reliably identified using routine susceptibility methods in local laboratories. Moreover, WGS represents a universal tool for epidemiological typing (EPI). These tests are currently performed at reference laboratories because the pathogens or diagnostic questions are relatively rare, which means that the costs of running these tests locally are prohibitive. In contrast, the fact that WGS is organism-independent would enable the necessary economies of scale in local laboratories.**

| **Species/disease** | **ID**[**^14^**](#_ENREF_14) | **Virulence determinants** [**^14-16^**](#_ENREF_14) | **Vaccine coverage**[**^16-18^**](#_ENREF_16) | **Significant**  **resistance mechanisms** | **EPI** | **Cases in England, Wales, and Northern Island in 2010^a^** |
| --- | --- | --- | --- | --- | --- | --- |
| *Acinetobacter baumannii* |  |  |  | ✓ | ✓ | 779 *Acinetobacter* spp.^b^  27% *A. baumannii* |
| *Campylobacter* |  |  |  |  | ✓ | 62,688 *Campylobacter* spp.^c^ |
| *Clostridium difficile* |  |  |  |  | ✓ | 23,215^d^ |
| *Enterobacter* |  |  |  | ✓ | ✓ | 2,037 *Enterobacter* spp.^b^  75% *E. cloacae* |
| Vancomycin-resistant *Enterorocci* |  |  |  | ✓ | ✓ | 541^b, d, e^ |
| *Escherichia coli* |  | ✓ |  | ✓ | ✓ | 27,062^b^  793 *E*. *coli* O157^c^ |
| *Haemophilus influenzae* |  |  | ✓ |  | ✓ | 546^c^ |
| *Klebsiella pneumoniae* |  | ✓ |  | ✓ | ✓ | 6,133 *Klebsiella* spp.^b^  74% *K. pneumoniae* |
| *Legionella pneumophila* |  |  |  |  | ✓ | 359^c^ |
| Meningococci |  |  | ✓ |  | ✓ | 929^b, c^  86% serogroup B |
| *Pseudomonas aeruginosa* |  | ✓ |  | ✓ | ✓ | 3,808 *Pseudomonas* spp.^b^  84% *P. aeruginosa* |
| Salmonellosis | ✓ |  |  | ✓ | ✓ | 9,133 *Salmonella* spp.^c^  27% Enteritidis  21% Typhimurium  6% Typhoidal  46% other serotypes |
| Shigellosis |  | ✓ |  | ✓ | ✓ | 1,747 *Shigella* spp.^c^  65% *S. sonnei*  28% *S. flexneri*  7% *S. boydii*, *S. dysenteriae* |
| MRSA |  | ✓ |  | ✓ | ✓ | 1,631^b,d^ |
| *Streptococcus agalactiae* |  |  |  |  | ✓ | 4,579 pyogenic streptococci^b^  35% *S. agalactiae* (group B streptococci)  34% *S. pyogenes* (group A streptocci)  31% group C+G streptococci |
| *Streptococcus pyogenes* |  | ✓ |  |  | ✓ |
| *Streptococcus pneumoniae* |  |  | ✓ |  | ✓ | 5,000-6,000^b^ |

^a^With the exception of the species covered by mandatory surveillance,[^4^](#_ENREF_4) these numbers represent an underestimate given that only a limited number of laboratories contribute to the voluntary surveillance scheme.[**^24-38^**](#_ENREF_24)

^b^Bacteremias/invasive disease only.

^c^England and Wales only.

^d^England only, mandatory surveillance.

^e^From 1 Oct 2009-30 Sep 2010

**Table S2**

Summary of the 17 reference strains and the 2 clinical isolates. The genomes sizes of these organisms ranged from 1.83 Mb for *Haemophilus influenzae* Rd KW20 to 6.26 Mb for *Pseudomonas aeruginosa* PAO1, which also had the highest GC content (67%) compared to just 28% for the plasmid of *Clostridium difficile* 630. Nine of the 17 species had an exclusively chromosomal genome, whereas the remaining species harboured up to five plasmids that ranged from 2 to 216 kb in size. The sequence data has been deposited at the European Nucleotide Archive (ENA) under the project code PRJEB4506.

| **Species** | **Comment** | **Chromosome/plasmid size (Mb/kb); %GC; accession number** | **ENA accession number of single-colony genome data** | **Source of strain^a^** |
| --- | --- | --- | --- | --- |
|
|
| ***Acinetobacter baumannii* ATCC 17978**[**^39^**](#_ENREF_39) |  | 3.98; 39; NC_009085.1  13.4; 36; NC_009083.1  11.3; 35; NC_009084.1 | ERS341395 | ATCC 17978 |
| ***Campylobacter jejuni* subsp. *jejuni* NCTC 11168**[**^40^**](#_ENREF_40) |  | 1.64; 31; NC_002163.1 | ERS341396 | NCTC 11168 |
| ***Clostridium difficile* 630**[**^41^**](#_ENREF_41) |  | 4.29; 29; NC_009089.1  7.9; 28; NC_008226.1 | ERS341397 | ATCC BAA-1382 |
| ***Enterobacter cloacae* subsp. *cloacae* type strain ATCC 13047**[**^42^**](#_ENREF_42) |  | 5.31; 55; NC_014121.1  199.6; 52; NC_014107.1  84.7; 47; NC_014108.1 | ERS341398 | ATCC 13047 |
| ***Enterococcus faecalis* V583**[**^43^**](#_ENREF_43) | Reference genomes for *E. faecium*, which accounts for the majority of vancomycin-resistant *Enterorocci* in the UK,[^44^](#_ENREF_44) were only published after the start of this study and we therefore used an *E. faecalis* reference isolate | 3.22; 38; NC_004668.1  66.3; 34; NC_004669.1  57.7; 34; NC_004671.1  18.0; 33; NC_004670.1 | ERS341399 | ATCC 700802 |
| ***Escherichia coli* str. K-12 substr. MG1655** | Laboratory strain | 4.64; 51; NC_000913.2 | ERS341400 | ATCC 700926 |
| ***Haemophilus influenzae* Rd KW20**[**^49^**](#_ENREF_49) | First genome of a free-living organism to be sequenced | 1.83; 38; NC_000907.1 | ERS341401 | ATCC 51907 |
| ***Klebsiella pneumoniae* subsp. *pneumoniae* MGH 78578**[**^50^**](#_ENREF_50) |  | 5.32; 57; NC_009648.1  175.9; 52; NC_009649.1  107.6; 53; NC_009650.1  88.6; 54; NC_009651.1  4.3; 41; NC_009652.1  3.5; 46; NC_009653.1 | ERS341402 | ATCC 700721 |
| **Clinical NDM-1-producing *Klebsiella pneumoniae*** | Isolated from a perinephric abscess on 17.5.2011 | Novel | ERS341403 | Cambridge PHE |
| ***Legionella pneumophila* subsp. *pneumophila* str. Philadelphia 1**[**^51^**](#_ENREF_51) | Representative of serogroup 1 which accounts for 97.6% of clinical isolates in UK[^52^](#_ENREF_52) | 3.40; 38; NC_002942.5 | ERS341404 | NCTC 11192 |
| ***Neisseria meningitidis* serogroup B strain MC58**[**^53^**](#_ENREF_53) | Most frequent serogroup in the UK. We included the isolate that was used to develop 4CmenB vaccine, which is currently being evaluated in clinical trials | 2.27; 52; NC_003112.2 | ERS341405 | ATCC BAA-335 |
| ***Pseudomonas aeruginosa* PAO1**[**^56^**](#_ENREF_56) |  | 6.26; 67; NC_002516.2 | ERS341406 | ATCC 47085 |
| ***Salmonella enterica* subsp. *enterica* serovar Enteritidis str. P125109**[**^57^**](#_ENREF_57) |  | 4.69; 52; NC_011294.1  39.9; 53; NC_003277.1^b^ | ERS341407 | NCTC 13349 |
| **Clinical *Salmonella enterica* subsp. *enterica* serovar Enteritidis** | Isolated from faeces on 14.12.2011 | Novel | ERS341408 | Cambridge PHE |
| ***Shigella sonnei* 53G**[**^58^**](#_ENREF_58) |  | 4.99; 51; NC_016822.1  215.8; 45; NC_016833.1  9.0; 40; NC_016834.1  5.2; 46; NC_016823.1  2.1; 47; NC_016824.1 | ERS341409 | Wellcome Trust Sanger Institute |
| ***Staphylococcus aureus* HO 5096 0412**[**^59^**](#_ENREF_59) | Sequence type 22, most frequent MRSA lineage in the UK[^60^](#_ENREF_60) | 2.83; 33; NC_017763.1  2.5; 31; NC_018969.1 | ERS341410 | Own stocks^c^ |
| ***Streptococcus agalactiae* 2603V/R**[**^61^**](#_ENREF_61) |  | 2.16; 36; NC_004116.1 | ERS341411 | ATCC BAA-611 |
| ***Streptococcus pneumoniae* TIGR4**[**^62^**](#_ENREF_62) |  | 2.16; 40; NC_003028.3 | ERS341412 | ATCC BAA-334 |
| ***Streptococcus pyogenes* M1**[**^63^**](#_ENREF_63) |  | 1.85; 39; NC_002737.1 | ERS341413 | ATCC 700294 |

^a^American Type Culture Collection (ATCC), purchased via LGC Standards (Teddington, UK); National Collection of Type Cultures (NCTC), Public Health England (Porton Down, UK).

^b^This strain of *Salmonella* contains a yet unpublished virulence plasmid that is similar to plasmid pSLT[^64^](#_ENREF_64) from *Salmonella enterica* subsp. *enterica* serovar Typhimurium str. LT2, which we used for this analysis (N. Thomson, Wellcome Trust Sanger Institute, personal communication).

^c^We have submitted this strain to NCTC under the accession 13616.

**Table S3**

Summary of standard culture conditions used to grow the 17 reference strains and 2 clinical isolates at the Cambridge Public Health England Clinical Microbiology and Public Health Laboratory. Depending on the sample type, selective, non-selective or indicator media are used to culture these pathogens. Unless otherwise stated, this involves incubation for 24 hours at 36 °C in air. The colony sizes obtained under these conditions varied markedly from as little as 0.3 mm for *Streptococcus pyogenes* M1 on UTI indicator medium, to several millimeters for some of the Gram-negative species on non-selective CBA medium. Nevertheless, single-colony WGS was possible for all of these species (Tables S4 and S5).

| **Species** | **Isolation site and culture media^a^** | | | | | | | | | |
| --- | --- | --- | --- | --- | --- | --- | --- | --- | --- | --- |
|  | **Blood and other sterile fluids** | **Urine** | **Wounds** | | **Faeces** | | | **Sputum** | **Nostrils and skin** | **Eyes, ears, nostrils, or throat** |
|  | CBA^b, c^ | UTI | CAP^c^ | CLED | XLD | CCEY^d^ | CCDA^e^ | BMPA^f^ | MRSA2 | CHOC^c^ |
| ***Acinetobacter baumannii* ATCC 17978**[**^39^**](#_ENREF_39) | ✓ | ✓ |  |  |  |  |  |  |  |  |
| ***Campylobacter jejuni* subsp. *jejuni* NCTC 11168**[**^40^**](#_ENREF_40) |  |  |  |  |  |  | ✓ |  |  |  |
| ***Clostridium difficile* 630**[**^41^**](#_ENREF_41) |  |  |  |  |  | ✓ |  |  |  |  |
| ***Enterobacter cloacae* subsp. *cloacae* type strain ATCC 13047**[**^42^**](#_ENREF_42) | ✓ | ✓ |  | ✓ |  |  |  |  |  |  |
| ***Enterococcus faecalis* V583**[**^43^**](#_ENREF_43) | ✓ | ✓ |  |  |  |  |  |  |  |  |
| ***Escherichia coli* str. K-12 substr. MG1655** | ✓ | ✓ |  |  |  |  |  |  |  |  |
| ***Haemophilus influenzae* Rd KW20**[**^49^**](#_ENREF_49) |  |  |  |  |  |  |  |  |  | ✓ |
| ***Klebsiella pneumoniae* subsp. *pneumoniae* MGH 78578**[**^50^**](#_ENREF_50) | ✓ | ✓ |  | ✓ |  |  |  |  |  |  |
| **Clinical NDM-1-producing *Klebsiella pneumoniae*** | ✓ |  |  |  |  |  |  |  |  |  |
| ***Legionella pneumophila* subsp. *pneumophila* str. Philadelphia 1**[**^51^**](#_ENREF_51) |  |  |  |  |  |  |  | ✓ |  |  |
| ***Neisseria meningitidis* serogroup B strain MC58**[**^53^**](#_ENREF_53) |  |  |  |  |  |  |  |  |  | ✓ |
| ***Pseudomonas aeruginosa* PAO1**[**^56^**](#_ENREF_56) | ✓ | ✓ |  | ✓ |  |  |  |  |  |  |
| ***Salmonella enterica* subsp. *enterica* serovar Enteritidis str. P125109**[**^57^**](#_ENREF_57) | ✓ | ✓ |  |  | ✓ |  |  |  |  |  |
| **Clinical *Salmonella enterica* subsp. *enterica* serovar Enteritidis** |  |  |  |  | ✓ |  |  |  |  |  |
| ***Shigella sonnei* 53G**[**^58^**](#_ENREF_58) | ✓ | ✓ |  |  | ✓ |  |  |  |  |  |
| ***Staphylococcus aureus* HO 5096 0412**[**^59^**](#_ENREF_59) | ✓ | ✓ | ✓ |  |  |  |  |  | ✓ |  |
| ***Streptococcus agalactiae* 2603V/R**[**^61^**](#_ENREF_61) | ✓ | ✓ | ✓ |  |  |  |  |  |  |  |
| ***Streptococcus pneumoniae* TIGR4**[**^62^**](#_ENREF_62) | ✓ |  | ✓ |  |  |  |  |  |  |  |
| ***Streptococcus pyogenes* M1**[**^63^**](#_ENREF_63) | ✓ | ✓ | ✓ |  |  |  |  |  |  |  |

^a^The following pre-prepared plates from Oxoid (Basingstoke, UK) were used: Columbia blood agar with horse blood (CBA), Brilliance urinary tract infection (UTI) clarity agar, Columbia colistin aztreonam (CAP) selective agar with horse blood, cystine lactose electrolyte deficient (CLED) agar, xylose lysine deoxycholate (XLD) agar, Campylobacter blood free selective agar (CCDA), Legionella BMPA selective agar, Brilliance MRSA2 agar, and Columbia agar with chocolate horse blood (CHOC). Brazier’s CCEY agar plates were prepared according to the manufacturer’s instructions (BioConnections, Wetherby, UK).

^b^CBA is used to subculture from positive blood-culture bottles.

^c^Incubated in 5% CO_2_.

^d^Incubated anaerobically.

^e^Incubated in microaerophilic conditions for 40 hours at 42 °C.

^f^Incubated for 72 hours in 5% CO_2_.

**Table S4**

Overview of the validation of the library preparation. For each of the 40 combinations of pathogen and culture conditions (Table S3), libraries were prepared in triplicate and shown to be sterile. A fourth library for each species done from CBA or equivalent medium, which is underlined, was sequenced to further verify the libraries, the results of which can be found in Table S5. For each condition the initial DNA concentration after lysis with beads is shown along with the concentration and peak of the final library.

| **Organism** | **Growth medium** | **Nextera kit** | **DNA conc. after bead lysis (ng/μL)** | **Library conc. (ng/μL)** | **Library peak (bp)** |
| --- | --- | --- | --- | --- | --- |
| ***Acinetobacter baumannii* ATCC 17978** | CBA | STD | 6.14 | 4.64 | 1327 |
|  |  |  | 13.5 | 4.26 | 1101 |
|  |  |  | 13.1 | 5.42 | 1126 |
|  |  |  | 6.7 | 5.66 | 941 |
|  | UTI | STD | 4.14 | 4.42 | 944 |
|  |  |  | 7.6 | 4.1 | 1021 |
|  |  |  | 4.96 | 4.72 | 892 |
| ***Campylobacter jejuni* subsp. *jejuni* NCTC 11168** | CCDA | STD | 5.42 | 2.94 | 1137 |
|  |  |  | 5.48 | 4.08 | 1304 |
|  |  |  | 4.88 | 3.78 | 1180 |
|  |  |  | 8.02 | 2.26 | 956 |
| ***Clostridium difficile* 630** | CCEY | XT | 2.36 | 0.708 | 598 |
|  |  |  | 0.986 | 0.706 | 674 |
|  |  |  | 1.14 | 0.698 | 728 |
|  |  |  | 1.48 | 1.59 | 575 |
| ***Enterobacter cloacae* subsp. *cloacae* type strain ATCC 13047** | CBA | STD | 4.36 | 5.28 | 1222 |
|  |  |  | 5.74 | 4.52 | 1150 |
|  |  |  | 4.66 | 5.08 | 998 |
|  |  |  | 3.74 | 2.86 | 1189 |
|  | UTI | STD | 4.46 | 2.78 | 1251 |
|  |  |  | 3.88 | 4.58 | 1135 |
|  |  |  | 9.56 | 2.54 | 1074 |
|  | CLED | STD | 9.24 | 3.48 | 1125 |
|  |  |  | 8.32 | 2.4 | 1114 |
|  |  |  | 7.72 | 2.02 | 1302 |
| ***Enterococcus faecalis* V583** | CBA | STD | 19.1 | 2.82 | 1198 |
|  |  |  | 39 | 4.14 | 1067 |
|  |  |  | 28 | 2.3 | 1350 |
|  |  |  | 12.9 | 6.6 | 1055 |
|  | UTI | XT | 3.02 | 1.76 | 901 |
|  |  |  | 3.98 | 1.85 | 919 |
|  |  |  | 6.12 | 2.28 | 874 |
| ***Escherichia coli* str. K-12 substr. MG1655** | CBA | STD | 9.34 | 4.08 | 1211 |
|  |  |  | 13.4 | 4.28 | 1314 |
|  |  |  | 19.5 | 3.94 | 1103 |
|  |  |  | 15.6 | 4.6 | 963 |
|  | UTI | STD | 11.4 | 2.08 | 460 |
|  |  |  | 9.86 | 3.4 | 506 |
|  |  |  | 7.36 | 1.61 | 921 |
| ***Haemophilus influenzae* Rd KW20** | CHOC | XT | 0.91 | 2.84 | 1239 |
|  |  |  | 1.53 | 2.32 | 1160 |
|  |  |  | 0.71 | 2.78 | 1314 |
|  |  |  | 26.2 | 0.738 | 1052 |
| ***Klebsiella pneumoniae* subsp. *pneumoniae* MGH 78578** | CBA | STD | 19.3 | 3.06 | 722 |
|  |  |  | 8.46 | 3.08 | 893 |
|  |  |  | 13.9 | 2.58 | 842 |
|  |  |  | 29.8 | 1.58 | 1142 |
|  | UTI | STD | 8.74 | 4.12 | 859 |
|  |  |  | 8.78 | 4.52 | 979 |
|  |  |  | 32.8 | 1.17 | 977 |
|  | CLED | STD | 8.22 | 3.72 | 1157 |
|  |  |  | 19.7 | 2.12 | 1045 |
|  |  |  | 10.4 | 1.6 | 1187 |
| **Clinical NDM-1-producing *K. pneumoniae*** | CBA | XT | 1.18 | 3.84 | 1265 |
|  |  |  | 2.12 | 4.3 | 1104 |
|  |  |  | 0.824 | 1.78 | 1289 |
|  |  |  | 1.87 | 2.28 | 1456 |
| ***Legionella pneumophila* subsp. *pneumophila* str. Philadelphia 1** | BMPA | XT | 0.99 | 4.64 | 877 |
|  |  |  | 1.34 | 3.2 | 907 |
|  |  |  | 0.648 | 2.72 | 882 |
|  |  |  | 1.44 | 4.22 | 864 |
| ***Neisseria meningitidis* serogroup B strain MC58** | CHOC | XT | 4.90 | 1.14 | 1160 |
|  |  |  | 4.54 | 0.71 | 1246 |
|  |  |  | 12.2 | 0.864 | 1274 |
|  |  |  | 42.8 | 1.49 | 1102 |
| ***Pseudomonas aeruginosa* PAO1** | CBA | STD | 16.1 | 3.84 | 1228 |
|  |  |  | 11.4 | 3.58 | 1342 |
|  |  |  | 13.8 | 4.38 | 1345 |
|  |  |  | 51.4 | 1.91 | 980 |
|  | UTI | STD | 15.1 | 2.58 | 983 |
|  |  |  | 7.8 | 1.41 | 1002 |
|  |  |  | 4.9 | 1.92 | 1064 |
|  | CLED | STD | 20 | 0.918 | 1265 |
|  |  |  | 8.82 | 2.14 | 1147 |
|  |  |  | 4.62 | 1.83 | 1040 |
| ***Salmonella enterica* subsp. *enterica* serovar Enteritidis str. P125109** | CBA | STD | 20.2 | 3.64 | 879 |
|  |  |  | 38.6 | 2.26 | 881 |
|  |  |  | 18.7 | 5.14 | 1054 |
|  |  |  | 47 | 3.22 | 513 |
|  | UTI | STD | 31 | 1.49 | 1105 |
|  |  |  | 35.2 | 2.3 | 1194 |
|  |  |  | 44.4 | 2.3 | 984 |
|  | XLD | STD | 15 | 3.4 | 930 |
|  |  |  | 16.7 | 3.34 | 528 |
|  |  |  | 15.2 | 3.1 | 528 |
| **Clinical *Salmonella enterica* subsp. *enterica* serovar Enteritidis** | XLD | STD | 15 | 2.74 | 982 |
|  |  |  | 7.84 | 2.6 | 1160 |
|  |  |  | 17.2 | 3.22 | 1046 |
|  |  |  | 18.8 | 3.1 | 577 |
| ***Shigella sonnei* 53G** | CBA | STD | 12.9 | 2.78 | 961 |
|  |  |  | 39.4 | 0.892 | 1162 |
|  |  |  | 15 | 1.66 | 1434 |
|  |  |  | 43.8 | 2 | 1833 |
|  | UTI | STD | 13.2 | 1.43 | 1047 |
|  |  |  | 15.1 | 0.94 | 1610 |
|  |  |  | 9 | 2.48 | 1496 |
|  | XLD | STD | 7.04 | 2.56 | 1057 |
|  |  |  | 23.6 | 1.37 | 969 |
|  |  |  | 4.02 | 1.85 | 975 |
| ***Staphylococcus aureus* HO 5096 0412** | CBA | STD | 21.8 | 4.02 | 1055 |
|  |  |  | 27 | 2.86 | 995 |
|  |  |  | 11.9 | 3.38 | 1232 |
|  |  |  | 26 | 3.76 | 1784 |
|  | UTI | STD | 8.1 | 3.9 | 1076 |
|  |  |  | 13.3 | 3.46 | 1222 |
|  |  |  | 8.2 | 3.94 | 1312 |
|  | CAP | STD | 20 | 2.68 | 983 |
|  |  |  | 22 | 2.6 | 1134 |
|  |  |  | 35.6 | 2.68 | 979 |
|  | MRSA2 | STD | 9.98 | 4.84 | 1159 |
|  |  |  | 8.18 | 4.42 | 1128 |
|  |  |  | 7.42 | 4.16 | 1092 |
| ***Streptococcus agalactiae* 2603V/R** | CBA | XT | 3.74 | 1.66 | 1402 |
|  |  |  | 4.6 | 1.85 | 1595 |
|  |  |  | 1.77 | 2.56 | 984 |
|  |  |  | 1.97 | 3.06 | 732 |
|  | UTI | XT | 2.3 | 2.3 | 950 |
|  |  |  | 1.99 | 1.58 | 943 |
|  |  |  | 1.92 | 3.64 | 893 |
|  | CAP | XT | 1.48 | 1.75 | 943 |
|  |  |  | 1.91 | 3.52 | 1048 |
|  |  |  | 2.18 | 1.74 | 1058 |
| ***Streptococcus pneumoniae* TIGR4** | CBA | XT | 0.414 | 0.928 | 1366 |
|  |  |  | 0.336 | 0.67 | 1487 |
|  |  |  | 0.274 | 2.4 | 977 |
|  |  |  | 1.04 | 1.24 | 981 |
|  | CAP | XT | 0.268 | 1.23 | 1152 |
|  |  |  | 0.63 | 1.67 | 931 |
|  |  |  | 0.868 | 1.09 | 985 |
| ***Streptococcus pyogenes* M1** | CBA | XT | 4.28 | 1.61 | 1017 |
|  |  |  | 3.80 | 2.14 | 994 |
|  |  |  | 2.54 | 2.74 | 968 |
|  |  |  | 4.38 | 2.2 | 742 |
|  | UTI | XT | 0.274 | 0.992 | 1182 |
|  |  |  | 1.41 | 3.68 | 1151 |
|  |  |  | 1.13 | 2.38 | 958 |
|  | CAP | XT | 3.28 | 1.32 | 1175 |
|  |  |  | 3.66 | 0.966 | 1057 |
|  |  |  | 3.84 | 1.07 | 994 |

**Table S5**

Summary of the sequencing results for one single-colony library for each of the 17 reference strains (the libraries in question are underlined in Table S4). The accession numbers for the reference chromosomes and plasmids can be found in Table S2 (the plasmids are shown in the same order). The variations in the percentage of the chromosomes or plasmids covered at >10x, which represents the minimum coverage commonly used to call SNPs,[^58^](#_ENREF_58) is due to homologous regions within the genome (for example, 99.91% of plasmid 2 of the *K. pneumoniae* reference strain is covered at >10x, when all sequence data is only mapped against this plasmid).[^65^](#_ENREF_65) The SNPs and indels relative to the published reference genomes likely represent errors in the reference genomes or true differences between variants of reference strains, as detected in previous studies that resequenced reference genomes.

| **Reference species** | **Part of genome** | **% unaligned reads** | **Depth** | **% uncovered** | **% coverage >10x** | **SNPs** | **Indels** |
| --- | --- | --- | --- | --- | --- | --- | --- |
| ***Acinetobacter baumannii* ATCC 17978** | Chr | 0.36 | 107.4 | 0.86 | 98.94 | 96 | 619 |
|  | Pl 1 |  | 479.7 | 0.01 | 99.89 | 0 | 1 |
|  | Pl 2 |  | 696.3 | 0.02 | 99.88 | 0 | 0 |
| ***Campylobacter jejuni* subsp. *jejuni* NCTC 11168** | Chr | 0.11 | 157.3 | 1.07 | 98.51 | 7 | 5 |
| ***Clostridium difficile* 630** | Chr | 0.46 | 129.6 | 2.19 | 96.71 | 14 | 5 |
|  | Pl |  | 1020 | 0.03 | 99.89 | 0 | 0 |
| ***Enterobacter cloacae* subsp. *cloacae* type strain ATCC 13047** | Chr | 0.25 | 103.5 | 1.47 | 97.58 | 34 | 117 |
|  | Pl 1 |  | 121.2 | 6.50 | 88.22 | 10 | 2 |
|  | Pl 2 |  | 141.5 | 0.00 | 99.98 | 1 | 1 |
| ***Enterococcus faecalis* V583** | Chr | 0.13 | 133.0 | 0.72 | 98.87 | 19 | 22 |
|  | Pl 1 |  | 220.9 | 2.27 | 91.97 | 0 | 0 |
|  | Pl 2 |  | 223.5 | 0.35 | 98.17 | 0 | 0 |
|  | Pl 3 |  | 287.1 | 0.52 | 94.53 | 0 | 0 |
| ***Escherichia coli* str. K-12 substr. MG1655** | Chr | 0.18 | 128.1 | 0.64 | 98.69 | 2 | 4 |
| ***Haemophilus influenzae* Rd KW20** | Chr | 0.39 | 234.3 | 2.00 | 97.71 | 294 | 250 |
| ***Klebsiella pneumoniae* subsp. *pneumoniae* MGH 78578** | Chr | 0.31 | 38.5 | 0.79 | 98.64 | 5 | 29 |
|  | Pl 1 |  | 47.1 | 17.21 | 76.99 | 0 | 2 |
|  | Pl 2 |  | 54.1 | 33.02 | 59.58 | 0 | 1 |
|  | Pl 3 |  | 56.0 | 3.24 | 86.80 | 0 | 0 |
|  | Pl 4 |  | 322.1 | 0.02 | 99.77 | 0 | 0 |
|  | Pl 5 |  | 253.5 | 0.06 | 99.86 | 0 | 0 |
| ***Legionella pneumophila* subsp. *pneumophila* str. Philadelphia 1** | Chr | 1.08 | 499.7 | 0.38 | 99.32 | 5 | 4 |
| ***Neisseria meningitidis* serogroup B strain MC58** | Chr | 0.88 | 101.4 | 5.26 | 93.18 | 4 | 26 |
| ***Pseudomonas aeruginosa* PAO1** | Chr | 0.51 | 204.3 | 0.45 | 99.33 | 24 | 35 |
| ***Salmonella enterica* subsp. *enterica* serovar Enteritidis str. P125109** | Chr | 0.64 | 62.2 | 0.93 | 98.90 | 1 | 0 |
|  | Pl**^a^** |  | 76.3 | 42.46 | 55.54 | 649 | 21 |
| ***Shigella sonnei* 53G** | Chr | 0.27 | 28.7 | 5.64 | 91.85 | 3 | 1 |
|  | Pl 1 |  | 50.0 | 10.73 | 78.52 | 0 | 0 |
|  | Pl 2^b^ |  | NA | NA | NA | NA | NA |
|  | Pl 3 |  | 564.4 | 0.04 | 99.75 | 0 | 0 |
|  | Pl 4 |  | 809.5 | 0.00 | 99.90 | 0 | 0 |
| ***Staphylococcus aureus* HO 5096 0412** | Chr | 0.25 | 79.0 | 1.28 | 98.25 | 0 | 2 |
|  | Pl |  | 893.8 | 0.04 | 99.72 | 0 | 0 |
| ***Streptococcus agalactiae* 2603V/R** | Chr | 0.26 | 120.1 | 2.13 | 97.07 | 7 | 2 |
| ***Streptococcus pneumoniae* TIGR4** | Chr | 0.43 | 176.9 | 1.72 | 97.50 | 40 | 21 |
| ***Streptococcus pyogenes* M1** | Chr | 0.38 | 285.6 | 1.56 | 98.03 | 176 | 28 |

^a^This strain of *Salmonella* contains a yet unpublished virulence plasmid that is similar to plasmid pSLT from *Salmonella enterica* subsp. *enterica* serovar Typhimurium str. LT2. (N. Thomson, Wellcome Trust Sanger Institute, personal communication). We included this plasmid as part of the analysis in order to demonstrate that the unusually high percentage of unaligned reads that occurs when the chromosome is analysed on its own corresponded to plasmid sequences.

^b^We sequenced this strain again using our single-colony protocol and also from DNA purified from liquid culture using the QIAamp DNA Mini Kit (Qiagen, Hilden, Germany)[^59^](#_ENREF_59) to confirm that this plasmid was not present in our stock of the reference strain.

**Table S6**

Carbapenamases used to confirm the genetic basis of carbapenem-resistance of the clinical *K. pneumoniae* isolate.

| **Gene** | **Accession number** |
| --- | --- |
| *bla*_KPC-2_ | AY034847 |
| *bla*_IMI-2_ | JN412066 |
| *bla*_NMC-A_ | Z21956 |
| *bla*_SME-1_ | JF974075 |
| *bla*_OXA-23_ | AJ132105 |
| *bla*_OXA-24_ | AF509241 |
| *bla*_OXA-48_ | AY236073 |
| *bla*_OXA-51_ | AJ309734 |
| *bla*_OXA-58_ | AY665723 |
| *bla*_AIM-1_ | AM998375 |
| *bla*_GES-1_ | AF156486 |
| *bla*_GIM-1_ | JF414726 |
| *bla*_IMP-1_ | S71932 |
| *bla*_KHM-1_ | AB364006 |
| *bla*_SIM-1_ | AY887066 |
| *bla*_SPM-1_ | AJ492820 |
| *bla*_NDM-1_ | JN872329 |
| *bla*_VIM-1_ | EF690696 |

**Table S7**

Previously sequenced *Salmonella* strains that were used for the analysis of our clinical isolate.

| **Subspecies** | **Serovar** | **Strain** | **Tree label** | **Reference(s)** |
| --- | --- | --- | --- | --- |
| arizonae | 62:z4,z23 | RSK2980 | arizonae | [^68^](#_ENREF_68) |
| enterica | Agona | SL483 | Agona | [^68^](#_ENREF_68) |
|  | Dublin^a^ | CT_02021853/SL477 | Dublin 1 | [^68^](#_ENREF_68) |
|  | Dublin^a^ | SD3246 | Dublin 2 | [^69^](#_ENREF_69) |
|  | Dublin^a^ | SL1438 | Dublin 3 | [^70^](#_ENREF_70) |
|  | Dublin^a^ | HWS51 | Dublin 4 | [^70^](#_ENREF_70) |
|  | Hadar | SL485 | Hadar | [^68^](#_ENREF_68) |
|  | Heidelberg | SL476 | Heidelberg | [^68^](#_ENREF_68) |
|  | Javiana | SL478 | Javiana | [^68^](#_ENREF_68) |
|  | Kentucky | SL475 | Kentucky | [^68^](#_ENREF_68) |
|  | Newport | SL254 | Newport | [^68^](#_ENREF_68) |
|  | Saintpaul | SARA23 | Saintpaul | [^68^](#_ENREF_68) |
|  | Schwarzengrund | CVM19633/SL473 | Schwarzengrund | [^68^](#_ENREF_68) |
|  | Virchow | SL491 | Virchow | [^68^](#_ENREF_68) |
|  | Weltevreden | SL484 | Weltevreden | [^68^](#_ENREF_68) |
|  | Choleraesuis | SC-B67 | Choleraesuis | [^71^](#_ENREF_71) |
|  | Gallinarum^a^ | 287/91 | Gallinarum 1 | [^57^](#_ENREF_57) |
|  | Gallinarum^a^ | SG9 | Gallinarum 2 | [^69^](#_ENREF_69) |
|  | Gallinarum^a^ | 9184 | Gallinarum 3 | [^70^](#_ENREF_70) |
|  | Paratyphi B | SPB7 | Paratyphi B | [^68^](#_ENREF_68) |
|  | Paratyphi C | RKS4594 | Paratyphi C | [^68^](#_ENREF_68) |
|  | Typhi | CT18 | Typhi | [^72^](#_ENREF_72) |
|  | Typhimurium | LT2 | Typhimurium | [^64^](#_ENREF_64) |
|  | Paratyphi A | ATCC 9150 | Paratyphi A | [^73^](#_ENREF_73) |
|  | Montevideo | 507440-20/217_Drain_Swab | Montevideo |  |
|  | Pollorum | RKS5078 | Pollorum 1 | [^76^](#_ENREF_76) |
|  | Pollorum | ATCC 9120 | Pollorum 2 | [^70^](#_ENREF_70) |
|  | Enteritidis | P125109/PT4 NCTC 13349 | Enteritidis reference | [^57^](#_ENREF_57) |
|  |  | LA5 | Enteritidis 1 | [^77^](#_ENREF_77) |
|  |  | CHS44 | Enteritidis 2 | [^70^](#_ENREF_70) |
|  |  | 56-3991 | Enteritidis 3 | [^70^](#_ENREF_70) |
|  |  | SE15-1 | Enteritidis 4 | [^70^](#_ENREF_70) |
|  |  | 629163 | Enteritidis 5 | [^70^](#_ENREF_70) |
|  |  | 22704 | Enteritidis 6 | [^70^](#_ENREF_70) |
|  |  | CDC 76-2651 | Enteritidis 7 | [^70^](#_ENREF_70) |
|  |  | CDC 81-2490 | Enteritidis 8 | [^70^](#_ENREF_70) |
|  |  | 13183-1 | Enteritidis 9 | [^70^](#_ENREF_70) |
|  |  | CDC 2010K-1445 | Enteritidis 10 | [^70^](#_ENREF_70) |

^a^We included several representatives of the serovars Dublin, Gallinarum, and Pollurum, given that these are closely related to Enteritidis.[^70^](#_ENREF_70) The individual strains are not visible at the resolution used in Figure 1.

**References**

1. Reuter S, Ellington MJ, Cartwright EJ et al. Rapid bacterial whole-genome sequencing to enhance diagnostic and public health microbiology. *JAMA Intern Med* 2013; **173**: 1397-404.

2. Hospital infection-working group of the Department of Health and Public Health Laboratory Service. Hospital infection control. Guidance on the control of infection in hospitals. 1995.

3. Johnson AP, Davies J, Guy R et al. Mandatory surveillance of methicillin-resistant *Staphylococcus aureus* (MRSA) bacteraemia in England: the first 10 years. *J Antimicrob Chemother* 2012; **67**: 802-9.

4. Wilson AP, Kiernan M. Recommendations for surveillance priorities for healthcare-associated infections and criteria for their conduct. *J Antimicrob Chemother* 2012; **67 Suppl 1**: i23-8.

5. Köser CU, Ellington MJ, Cartwright EJ et al. Routine use of microbial whole genome sequencing in diagnostic and public health microbiology. *PLoS Pathog* 2012; **8**: e1002824.

6. Köser CU, Bryant JM, Becq J et al. Whole-genome sequencing for rapid susceptibility testing of *M. tuberculosis*. *N Engl J Med* 2013; **369**: 290-2.

7. Jorgensen JH, Ferraro MJ. Antimicrobial susceptibility testing: a review of general principles and contemporary practices. *Clin Infect Dis* 2009; **49**: 1749-55.

8. Rice LB. The clinical consequences of antimicrobial resistance. *Curr Opin Microbiol* 2009; **12**: 476-81.

9. Nordmann P, Poirel L, Walsh TR et al. The emerging NDM carbapenemases. *Trends Microbiol* 2011; **19**: 588-95.

10. Didelot X, Bowden R, Wilson DJ et al. Transforming clinical microbiology with bacterial genome sequencing. *Nat Rev Genet* 2012; **13**: 601-12.

11. Livermore DM, Andrews JM, Hawkey PM et al. Are susceptibility tests enough, or should laboratories still seek ESBLs and carbapenemases directly? *J Antimicrob Chemother* 2012; **67**: 1569-77.

12. Mosammaparast N, McAdam AJ, Nolte FS. Molecular testing for infectious diseases should be done in the clinical microbiology laboratory. *J Clin Microbiol* 2012; **50**: 1836-40.

13. van Belkum A, Durand G, Peyret M et al. Rapid clinical bacteriology and its future impact. *Annals of laboratory medicine* 2013; **33**: 14-27.

14. Health Protection Agency. Laboratory of gastrointestinal pathogens - user manual (July 2012). http://www.hpa.org.uk/webc/HPAwebFile/HPAweb_C/1247816548297 (accessed 7.1.2013).

15. Health Protection Agency. Department of healthcare associated infection and antibiotic resistance - user manual (effective date: 26.07.11). http://www.hpa.org.uk/webc/HPAwebFile/HPAweb_C/1194947406124 (accessed 7.1.2013).

16. Health Protection Agency. Respiratory and systemic infection laboratory - user manual (effective date: 28.11.2011). http://www.hpa.org.uk/webc/HPAwebFile/HPAweb_C/1194947411598 (accessed 7.1.2013).

17. Health Protection Agency. Meningococcal reference unit - user manual (April 2012). http://www.hpa.org.uk/webc/HPAwebFile/HPAweb_C/1194947367872 (accessed 7.1.2013).

18. Lucidarme J, Comanducci M, Findlow J et al. Characterization of *fHbp*, *nhba* (*gna2132*), *nadA*, *porA*, and sequence type in group B meningococcal case isolates collected in England and Wales during January 2008 and potential coverage of an investigational group B meningococcal vaccine. *Clin Vaccine Immunol* 2010; **17**: 919-29.

19. Health Protection Agency. National resistance alert: potentially transferable linezolid resistance in *Enterococcus faecium* in the UK (September 2012). http://www.hpa.org.uk/webc/HPAwebFile/HPAweb_C/1317135991530 (accessed 7.1.2013).

20. Pichon B, Hill R, Laurent F et al. Development of a real-time quadruplex PCR assay for simultaneous detection of nuc, Panton-Valentine leucocidin (PVL), *mecA* and homologue *mecA*LGA251. *J Antimicrob Chemother* 2012; **67**: 2338-41.

21. Health Protection Agency. Advice on carbapenemase producers: recognition, infection control and treatment. http://www.hpa.org.uk/webc/HPAwebFile/HPAweb_C/1294740725984 (accessed 7.1.2013).

22. Fawley WN, Wilcox MH, Clostridium difficile Ribotyping Network for England and Northern Ireland. An enhanced DNA fingerprinting service to investigate potential *Clostridium difficile* infection case clusters sharing the same PCR ribotype. *J Clin Microbiol* 2011; **49**: 4333-7.

23. Wilcox MH, Shetty N, Fawley WN et al. Changing epidemiology of *Clostridium difficile* infection following the introduction of a national ribotyping-based surveillance scheme in England. *Clin Infect Dis* 2012; **55**: 1056-63.

24. Health Protection Agency. *Acinetobacter* spp. bacteraemia, England, Wales, and Northern Ireland: 2006 to 2010. http://www.hpa.org.uk/webc/HPAwebFile/HPAweb_C/1317131514188 (accessed 7.1.2013).

25. Health Protection Agency. Laboratory reports of Campylobacter sp in England and Wales 2000-2011. http://www.hpa.org.uk/Topics/InfectiousDiseases/InfectionsAZ/Campylobacter/EpidemiologicalData/campyDataEw/ (accessed 7.11.13).

26. Health Protection Agency. Quarterly epidemiological commentary: Mandatory MRSA, MSSA and *E. coli* bacteraemia, and *C. difficile* infection data (up to April–June 2012). http://www.hpa.org.uk/webc/HPAwebFile/HPAweb_C/1317137340354. (accessed 7.1.13).

27. Health Protection Agency. *Klebsiella*, *Enterobacter*, *Serratia*, and *Citrobacter* bacteraemia, England, Wales, and Northern Ireland: 2007-2011. http://www.hpa.org.uk/webc/HPAwebFile/HPAweb_C/1317136602321 (accessed 7.1.2013).

28. Health Protection Agency. Annual counts of glycopeptide resistant enterococcal bacteraemia (October 2003 - September 2011). http://www.hpa.org.uk/webc/HPAwebFile/HPAweb_C/1278944230968 (accessed 7.1.2013).

29. Health Protection Agency. *Escherichia coli* bacteraemia in England, Wales and Northern Ireland, 2007-2011. http://www.hpa.org.uk/webc/HPAwebFile/HPAweb_C/1317134482059. (accessed 7.1.2013).

30. Health Protection Agency. *E. coli* O157 annual totals. http://www.hpa.org.uk/web/HPAweb&HPAwebStandard/HPAweb_C/1249113624846 (accessed 7.1.2013).

31. Health Protection Agency. Laboratory reports of *Haemophilus influenzae* infection by serotype and year: England and Wales, 1990 to 2010. http://www.hpa.org.uk/Topics/InfectiousDiseases/InfectionsAZ/HaemophilusInfluenzaeTypeB/EpidemiologicalData/haem_DataAllSerotypes (accessed 7.1.2013).

32. Health Protection Agency. Legionnaires' disease in residents of England and Wales - nosocomial, travel or community acquired cases, 1980-2011. http://www.hpa.org.uk/web/HPAweb&HPAwebStandard/HPAweb_C/1195733748327 (accessed 7.1.2013).

33. Health Protection Agency. Invasive meningococcal infections laboratory reports, England and Wales by capsular group & calendar year, 1998-2012. http://www.hpa.org.uk/webc/HPAwebFile/HPAweb_C/1317136087786 (accessed 7.1.2013).

34. Health Protection Agency. *Pseudomonas* spp. and *Stenotrophomonas maltophilia* bacteraemia in England, Wales, and Northern Ireland, 2007 to 2011. http://www.hpa.org.uk/webc/HPAwebFile/HPAweb_C/1317135098542 (accessed 7.1.2013).

35. Health Protection Agency. Salmonella by serotype. http://www.hpa.org.uk/Topics/InfectiousDiseases/InfectionsAZ/Salmonella/EpidemiologicalData/salmDataHuman (accessed 7.1.2013).

36. Health Protection Agency. *Shigella* spp. laboratory reports of faecal isolates reported to the Health Protection Agency Centre for Infections England and Wales, 1992-2010. http://www.hpa.org.uk/Topics/InfectiousDiseases/InfectionsAZ/Shigella/EpidemiologicalData (accessed 7.1.2013).

37. Health Protection Agency. Pyogenic and non-pyogenic streptococcal bacteraemia, England, Wales and Northern Ireland: 2011. http://www.hpa.org.uk/webc/HPAwebFile/HPAweb_C/1317136996075 (accessed 7.1.2013).

38. Health Protection Agency. General information on pneumococcal disease. http://www.hpa.org.uk/Topics/InfectiousDiseases/InfectionsAZ/Pneumococcal/GeneralInformationPneumococcal/pneumoGeneralInformation/ (accessed 7.1.2013).

39. Smith MG, Gianoulis TA, Pukatzki S et al. New insights into *Acinetobacter baumannii* pathogenesis revealed by high-density pyrosequencing and transposon mutagenesis. *Genes Dev* 2007; **21**: 601-14.

40. Parkhill J, Wren BW, Mungall K et al. The genome sequence of the food-borne pathogen *Campylobacter jejuni* reveals hypervariable sequences. *Nature* 2000; **403**: 665-8.

41. Sebaihia M, Wren BW, Mullany P et al. The multidrug-resistant human pathogen *Clostridium difficile* has a highly mobile, mosaic genome. *Nat Genet* 2006; **38**: 779-86.

42. Ren Y, Zhou Z, Guo X et al. Complete genome sequence of *Enterobacter cloacae* subsp. *cloacae* type strain ATCC 13047. *J Bacteriol* 2010; **192**: 2463-4.

43. Paulsen IT, Banerjei L, Myers GS et al. Role of mobile DNA in the evolution of vancomycin-resistant *Enterococcus faecalis*. *Science* 2003; **299**: 2071-4.

44. Werner G, Coque TM, Hammerum AM et al. Emergence and spread of vancomycin resistance among enterococci in Europe. *Euro Surveill* 2008; **13**: pii: 19046.

45. Lam MM, Seemann T, Bulach DM et al. Comparative analysis of the first complete *Enterococcus faecium* genome. *J Bacteriol* 2012; **194**: 2334-41.

46. Qin X, Galloway-Pena JR, Sillanpaa J et al. Complete genome sequence of *Enterococcus faecium* strain TX16 and comparative genomic analysis of *Enterococcus faecium* genomes. *BMC Microbiol* 2012; **12**: 135.

47. Blattner FR, Plunkett G, 3rd, Bloch CA et al. The complete genome sequence of *Escherichia coli* K-12. *Science* 1997; **277**: 1453-62.

48. Riley M, Abe T, Arnaud MB et al. *Escherichia coli* K-12: a cooperatively developed annotation snapshot-2005. *Nucleic Acids Res* 2006; **34**: 1-9.

49. Fleischmann RD, Adams MD, White O et al. Whole-genome random sequencing and assembly of *Haemophilus influenzae* Rd. *Science* 1995; **269**: 496-512.

50. Seo JH, Hong JS, Kim D et al. Multiple-omic data analysis of *Klebsiella pneumoniae* MGH 78578 reveals its transcriptional architecture and regulatory features. *BMC Genomics* 2012; **13**: 679.

51. Chien M, Morozova I, Shi S et al. The genomic sequence of the accidental pathogen *Legionella pneumophila*. *Science* 2004; **305**: 1966-8.

52. Harrison TG, Afshar B, Doshi N et al. Distribution of *Legionella pneumophila* serogroups, monoclonal antibody subgroups and DNA sequence types in recent clinical and environmental isolates from England and Wales (2000-2008). *Eur J Clin Microbiol Infect Dis* 2009; **28**: 781-91.

53. Tettelin H, Saunders NJ, Heidelberg J et al. Complete genome sequence of *Neisseria meningitidis* serogroup B strain MC58. *Science* 2000; **287**: 1809-15.

54. Pizza M, Scarlato V, Masignani V et al. Identification of vaccine candidates against serogroup B meningococcus by whole-genome sequencing. *Science* 2000; **287**: 1816-20.

55. Bai X, Findlow J, Borrow R. Recombinant protein meningococcal serogroup B vaccine combined with outer membrane vesicles. *Expert Opin Biol Ther* 2011; **11**: 969-85.

56. Stover CK, Pham XQ, Erwin AL et al. Complete genome sequence of *Pseudomonas aeruginosa* PAO1, an opportunistic pathogen. *Nature* 2000; **406**: 959-64.

57. Thomson NR, Clayton DJ, Windhorst D et al. Comparative genome analysis of *Salmonella* Enteritidis PT4 and *Salmonella* Gallinarum 287/91 provides insights into evolutionary and host adaptation pathways. *Genome Res* 2008; **18**: 1624-37.

58. Holt KE, Baker S, Weill FX et al. *Shigella sonnei* genome sequencing and phylogenetic analysis indicate recent global dissemination from Europe. *Nat Genet* 2012; **44**: 1056-9.

59. Köser CU, Holden MT, Ellington MJ et al. Rapid whole-genome sequencing for investigation of a neonatal MRSA outbreak. *N Engl J Med* 2012; **366**: 2267-75.

60. Ellington MJ, Hope R, Livermore DM et al. Decline of EMRSA-16 amongst methicillin-resistant *Staphylococcus aureus* causing bacteraemias in the UK between 2001 and 2007. *J Antimicrob Chemother* 2010; **65**: 446-8.

61. Tettelin H, Masignani V, Cieslewicz MJ et al. Complete genome sequence and comparative genomic analysis of an emerging human pathogen, serotype V *Streptococcus agalactiae*. *Proc Natl Acad Sci U S A* 2002; **99**: 12391-6.

62. Tettelin H, Nelson KE, Paulsen IT et al. Complete genome sequence of a virulent isolate of *Streptococcus pneumoniae*. *Science* 2001; **293**: 498-506.

63. Ferretti JJ, McShan WM, Ajdic D et al. Complete genome sequence of an M1 strain of *Streptococcus pyogenes*. *Proc Natl Acad Sci U S A* 2001; **98**: 4658-63.

64. McClelland M, Sanderson KE, Spieth J et al. Complete genome sequence of *Salmonella enterica* serovar Typhimurium LT2. *Nature* 2001; **413**: 852-6.

65. Cahill MJ, Köser CU, Ross NE et al. Read length and repeat resolution: exploring prokaryote genomes using next-generation sequencing technologies. *PLoS One* 2010; **5**: e11518.

66. Köser CU, Niemann S, Summers DK et al. Overview of errors in the reference sequence and annotation of *Mycobacterium tuberculosis* H37Rv, and variation amongst its isolates. *Infect Genet Evol* 2012; **12**: 807-10.

67. Ribeiro FJ, Przybylski D, Yin S et al. Finished bacterial genomes from shotgun sequence data. *Genome Res* 2012; **22**: 2270-7.

68. Fricke WF, Mammel MK, McDermott PF et al. Comparative genomics of 28 *Salmonella enterica* isolates: evidence for CRISPR-mediated adaptive sublineage evolution. *J Bacteriol* 2011; **193**: 3556-68.

69. Richardson EJ, Limaye B, Inamdar H et al. Genome sequences of *Salmonella enterica* serovar Typhimurium, Choleraesuis, Dublin, and Gallinarum strains of well- defined virulence in food-producing animals. *J Bacteriol* 2011; **193**: 3162-3.

70. Allard MW, Luo Y, Strain E et al. On the evolutionary history, population genetics and diversity among isolates of *Salmonella* Enteritidis PFGE pattern JEGX01.0004. *PLoS One* 2013; **8**: e55254.

71. Chiu CH, Tang P, Chu C et al. The genome sequence of *Salmonella enterica* serovar Choleraesuis, a highly invasive and resistant zoonotic pathogen. *Nucleic Acids Res* 2005; **33**: 1690-8.

72. Parkhill J, Dougan G, James KD et al. Complete genome sequence of a multiple drug resistant *Salmonella enterica* serovar Typhi CT18. *Nature* 2001; **413**: 848-52.

73. McClelland M, Sanderson KE, Clifton SW et al. Comparison of genome degradation in Paratyphi A and Typhi, human-restricted serovars of *Salmonella enterica* that cause typhoid. *Nat Genet* 2004; **36**: 1268-74.

74. Lienau EK, Strain E, Wang C et al. Identification of a salmonellosis outbreak by means of molecular sequencing. *N Engl J Med* 2011; **364**: 981-2.

75. Allard MW, Luo Y, Strain E et al. High resolution clustering of *Salmonella enterica* serovar Montevideo strains using a next-generation sequencing approach. *BMC Genomics* 2012; **13**: 32.

76. Feng Y, Xu HF, Li QH et al. Complete genome sequence of *Salmonella enterica* serovar pullorum RKS5078. *J Bacteriol* 2012; **194**: 744.

77. Grépinet O, Rossignol A, Loux V et al. Genome sequence of the invasive *Salmonella enterica* subsp. *enterica* serotype Enteritidis strain LA5. *J Bacteriol* 2012; **194**: 2387-8.
